# Preparation and characterization of coconut shell activated carbon supported titanium dioxide nanocomposite (CSAC-P-TiO_2—_NC)

**DOI:** 10.1016/j.mex.2026.103862

**Published:** 2026-03-17

**Authors:** Adewumi Oluwasogo Dada, Oladipo A Christianah, Olowojoba-Ozoh M Odufa, Kehinde Shola Obayomi, Ajanaku O. Christiana, Sook K Chang, Ling S Wong, Lee D Wilson

**Affiliations:** a Industrial Chemistry Programme, Department of Physical Sciences, Landmark University, P.M.B.1001, Omu-Aran, Kwara State, Nigeria; b Landmark University SDG 6: Clean Water and Sanitation, P.M.B.1001, OmuAran, Kwara State, Nigeria; c Landmark University SDG 7: Affordable and Clean Energy, P.M.B.1001, OmuAran, Kwara State, Nigeria; d Landmark University SDG 12: Responsible Consumption and Production, P.M.B.1001, OmuAran, Kwara State, Nigeria; e Landmark University SDG 9: Industry, Innovation and Infrastructure, P.M.B.1001, Omu-Aran, Kwara State, Nigeria; f Institute for Sustainable Industries and Liveable Cities, Victoria University, PO Box 14428, Melbourne, Victoria 8001, Australia; g Faculty of Health and Life Sciences, INTI International University, Persiaran Perdana BBN, Putra Nilai, Nilai, Negeri Sembilan 71800, Malaysia; h Department of Chemistry, University of Saskatchewan, 110 Science Place, Saskatoon, Saskatchewan S7N 5C9, Canada

**Keywords:** Biomass-derived materials, Pollutants, Titanium nanocomposites, Morphological characterization, Eco-friendly processes

## Abstract

There is an ever-increasing need in the engineering and development of cost-effective and efficient carbon decorated nano-composites due to highly expensive challenges from activated carbon and its affordability in the developing region of the world. This study was posed to investigate the preparation and characterization of coconut shell activated carbon decorated titanium dioxide nanocomposites (CSAC-P-TiO_2—_NC). The physicochemical characterization of the developed material, as revealed from the results, showed the optimum pH to be 6.57, pHpzc value of 4.8, and 5.3% ash content. Spectroscopic data revealed thermal stability of the nanocomposite up to 900 °C without significant loss in weight. Availability of a wide surface area was obtained as revealed by the BET results of 85.130 m^2^/g. FTIR result revealed the various functional groups present in the nanocomposite with the characteristic peak of Ti-O stretch at 799 cm^-1^. XRD patterns further confirmed the anatase phase of titanium dioxide crystalline mesoporous structure of the nanocomposite, having a characteristic diffraction peak at 2θ = 25.3 ° Scanning Electron Microscopy (SEM) revealed the morphological view of the nanostructure, containing numerous wide pore spaces that were observed on its surface. Transmission Electron Microscopy (TEM) revealed a fine anatase crystalline mesoporous structure of the titanium dioxide nanocomposites with spherical shape and 55–60 nm size. Thermogravimetric Analysis (TGA) showed a high thermal stability up to 900 °C. XPS measurement confirms the successful formation of carbon decorated CSAC-P-TiO_2—_NC containing Titanium-based oxide with lattice oxygen and carbon showing characteristic binding energies for carbon (284.8 eV and 289.2 eV), oxygen (530.6 eV), and titanium (459.4 eV and 465.0 eV). The physicochemical and spectroscopic analyses demonstrated that titanium dioxide nanoparticles were successfully incorporated into the phosphoric acid developed coconut shell activated carbon to yield the carbon-based decorated titanium dioxide nanocomposite. These characteristics properties revealed the potential of CSAC-P-TiO_2—_NC as a promising and sustainable low-cost potential adsorbent and photocatalyst recommended for pollutant removal for wastewater remediation.

The highlights from this article are:

• Development of sustainable and eco-friendly coconut shell activated carbon decorated titanium dioxide nanocomposites (CSAC-P-TiO_2—_NC) via hydrothermal technique
• Phosphoric acid activation improved and enhanced the porosity and surface area of the carbon decorated titanium nanocomposites, determined by BET, promoting TiO_2_ dispersion
• Successful integration of TiO_2_ onto CSAC matrix was confirmed by spectroscopic, structural, and morphological analyses (XRD, FTIR, SEM-EDX, HR-TEM and XPS)

## Graphical abstract

**Figure:**
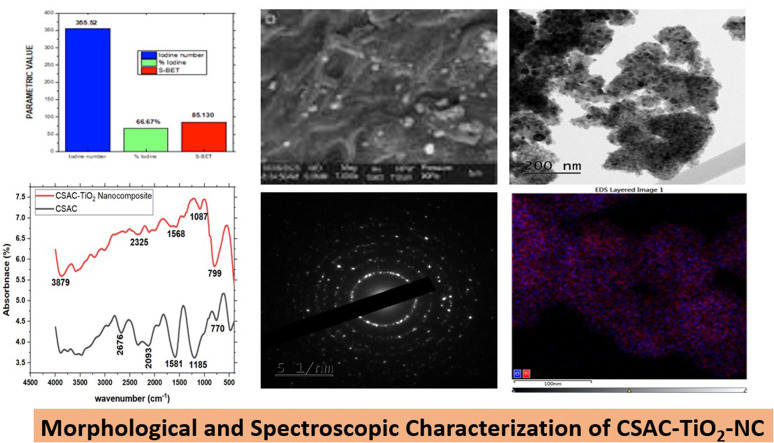

Graphical Abstract of CSAC-P-TiO_2—_NC

**Specifications Table**

**Table:** 

Subject area	Chemistry
**More specific subject area**	Nanotechnology & Material Chemistry
**Name of your method**	Hydrothermal synthetic route of Preparation of Carbon supported Titanium Nanocomposite, physicochemical and spectroscopic characterization
**Name and reference of original method**	Adewumi Oluwasogo Dada, Abosede Adejumoke Inyinbor, Blessing Enyojo Tokula, Abiodun Ajibola Bayode, Kehinde Shola Obayomi, Christiana Oluwatoyin Ajanaku, Folahan Amoo Adekola, Kolawole Oluseyi Ajanaku, Ujjwal Pal (2024). Zinc oxide decorated plantain peel activated carbon for adsorption of cationic malachite green dye: Mechanistic, kinetics and thermodynamics modeling, Environmental Research, Volume 252, Part 3, 2024, 119,046, https://doi.org/10.1016/j.envres.2024.119046. (https://www.sciencedirect.com/science/article/pii/S0013935124009502)
**Resource availability**	Available within the article

## Background

The interest of researchers in nanotechnology and its application across numerous industries has increased in recent years. This is attributed to the many distinct characteristics of very wide surfaces, numerous binding sites, and easily modifiable surfaces, leading to the possibility and ease of novel and improved nanoparticles development [1]. Pollution, especially of the environment and water bodies have generated a lot of research problems because many industries and processes use a large volume of water daily and also generate a high amount of wastewater, which is often discarded untreated or poorly treated into water bodies [1,2]. Several pollutants arising from dyes, heavy metals, pharmaceuticals, and herbicides, which are endocrine-disruptive in nature, are inherently present in the water body, and unfortunately, these endocrine EDCs are mostly not biodegradable. They bio-accumulate or combine with atmospheric elements to form stable and toxic substances [3] that are hazardous to human and animal lives [4,5]. An immediate observation of polluted water and the environment is discoloration and a pungent smell [6] . As a result of indiscriminate release of polluted wastewaters into water bodies, human life is at risk of various diverse diseases which may be mild, severe and sometimes leading to death [7]. Over the years, different materials such as clay, zeolites, activated carbon [8,9] to mention but a few, and methods like ion exchange, ozonation, membrane separation and photodegradation have been developed and prepared for use in the removal of wastes from water bodies [10]. The use of these materials and methods has recorded some level of success; however, concerns about the cost, unique properties, availability and release of secondary pollutants have militated against their efficiencies [11].

Activated carbon has found a wide application owing to its unique exceptional properties as regards high surface area, porosity, and functionality. However, it also exhibits limitations arising from selectivity, lower adsorption capacity for some specific pollutants and deterioration in terms of complex matrices. Similarly, unsupported and bare nanoparticles like metal oxide and noble metal nanoparticles, though they possess high intrinsic reactivity and abundant active sites, suffer some limitations arising from agglomeration, aggregation, leaching, poor stability and reusability disadvantages [[12], [13], [14], [15]]. The intrinsic drawbacks of both bare AC and unsupported nanoparticles (NPs) are addressed by the synergistic benefits of carbon-based supported nanocomposites, in which single nanoparticles are immobilized on a carbonaceous support like activated carbon, graphene, or carbon nanotubes. Specifically, the prompting on the development of carbon decorated titanium nanocomposite (CSAC-P-TiO2-NC) was based on the advantages and benefits of developing a carbon-supported nanocomposite as reported in literature such as: increased density of reactive sites and the development of several interaction mechanisms (such as electrostatic, and π–π interactions) beyond those found in bare AC alone are two benefits of adding nanoparticles to a high-surface-area carbon support nanocomposite [13,14,16]; stability and improved dispersion, eradicating the challenge of agglomeration [17]; ease of recovery and reusability making it easier to separate, reducing or eradicating the risk of environmental leaching [18]; and overall, the carbon supported nanocomposite enhances the tunability of physicochemical properties which could help in tailoring the synthesized nanocomposites based on specificity [19] hence the supporting motivation for this study. Advantageously, the carbon source from this study was derived from extremely low-cost agro-waste, readily available and sustainable coconut shell and to the best of our knowledge, there are limited extensive studies on the developed CSAC-P-TiO_2—_NC vis-à-vis their spectroscopic, morphological and elemental distribution characterization in terms of BET, XRD, FTIR, TGA, XPS, SEM-EDX and TEM as well as physicochemical parameter properties, which form the main scope and focus of this study. The preparation of nanocomposites from ubiquitous agro-waste offers a suitable and sustainable route for the development of materials. In future applications, the carbon-decorated titanium oxide nanocomposite developed from this study is expected to find effective and efficient applications in wastewater treatment and environmental remediation [9,20].

## Method details

### Materials and reagents used

Analytical grade reagents from Sigma Aldrich (USA) were used without further modification. Some of the reagents are: orthophosphoric acid (H_3_PO_4_), (CAS No: 7664 – 38 - 2), HCl (Hydrochloric acid) (CAS: 7697 – 37 - 2), sodium hydroxide (NaOH) (Carlo Erba CAS: 1310–73–2), magnetic stirrer (Bante MS300), titanium isopropoxide, pH meter (Milwaukee instrument: MW102 PRO 2-in-1 pH and Temperature meter), oven (GENLAB N30C), and furnace (Searchtech SX-5–12). The raw coconut shells were collected from Omu-Aran local market, Longitude (5.1026 ° E) and Latitude (8.1386 ° N), Kwara State, Nigeria.

#### Sample pretreatment and carbonization

The coconut shells were sorted, washed and dried to remove unwanted particles and dirt, followed by carbonization at 400 °C for 1 hour. The carbonized coconut shells were turned into fine powder by grinding with a mortar and pestle and sieving with a 0.15 mm sieve before being labelled as CS [9,12].

a. **Chemical Activation of Carbonized Coconut Shell**

Orthophosphoric acid was used to chemically activate the CS as follows: in a beaker containing 1000 mL of 0.5 M H_3_PO_4_, 1000 g of carbonized CS was weighed and added, heat was applied until the mixture turned into a paste, which was placed in a furnace set at 400 °C to heat for 1 hr. This process turned the carbonized CS into activated carbon, which was allowed to cool before thoroughly rinsing with distilled water to get a neutral pH of 7.0. The activated carbon was dried using an oven for 2 h at 100 °C and kept in an air-tight container. This was further dried in an oven before packing and labelling as CSAC-P [9].

b. **Titanium dioxide nanoparticle synthesis**

The hydrothermal method was used to prepare the nanoparticles. 30 mL of ethanol was used to dissolve 10 mL of titanium tetraisopropoxide while stirring continuously for 30 min in a magnetic stirrer, a few drops of distilled water was added to form a white dispersion medium, a filter paper was used to filter the precipitate formed before washing with distilled water 4–5 times and drying in an oven for 5 h at 100 °C. This was followed by calcination and further dying of the formed powder for 3 h at 500 °C hours, allowed to cool, labelled as TiO_2_ – NPs and stored [12,15].

c. **Preparation of Carbon-supported TiO_2_ Nanocomposite**

**Scheme 1:**
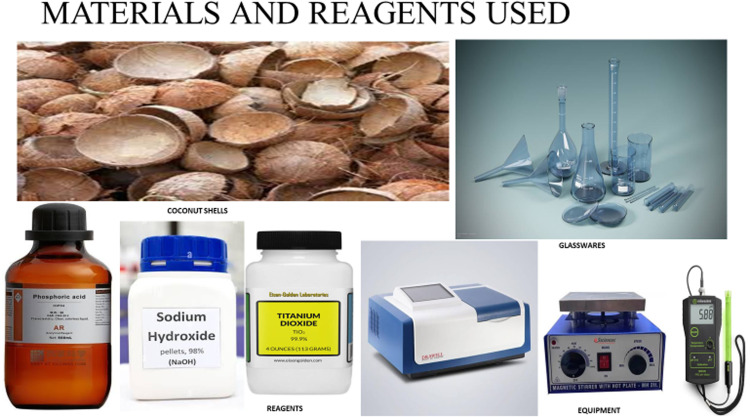
Different materials, reagent and equipment used in the preparation of CSAC-P & CSAC-P-TiO_2—_NC.

A slight modification of the method reported in the literature was used to prepare the nanocomposite. To obtain the desired nanocomposites, a 5:1 of CSAC and TiO_2_ – NPs was used for the preparation. 100 mL of distilled water was used to dissolve 1.0 g of CSAC while 20 mL of distilled water was used to dissolve 1.0 g of CSAC and TiO_2_ – NPs and ultrasonicated for 30 min. The CSAC and TiO_2_ – NPs solutions were mixed while sonicating for 30 min. Filtration with a filter paper was carried out on the suspension. The composite was dried at 100 °C overnight in an oven. After cooling, it was labelled as CSAC-P-TiO2-NC and stored appropriately, [9]. (Scheme 1)

**Physicochemical Characterization of Prepared Nanocomposite:**

i. **Determination of pH:** 100 mL of distilled water was measured into a beaker, 1.0 g CSAC-P-TiO_2—_NC was added to it, the beaker was allowed to boil for 5 min in a hot plate before adding another 100 mL of distilled water, the resulting solution was left to cool and the pH value was taken with a pH meter according to [21].
ii. **Bulk Density**: An empty measuring cylinder of 10 mL capacity was measured. The weight of the measuring cylinder was measured again after filling it up CSAC-P-TiO_2—_NC and tapping gently to expel air trapped within particles [10]. The differences in the weights of the measuring cylinder when empty and after being packed with the CSAC-P-TiO_2—_NC was determined [1], the bulk density was computed with the Eq. (1):
  (1) $$\text{Bulk}\;\text{Density}=\frac{w2-w1}{v}\times 100$$

Where: w_2=_the weight of the measuring cylinder packed filled with CSAC-P-TiO_2—_NC; w_1_= the weight of the empty cylinder_;_ and *v* = volume of the cylinder.

iii. **Moisture Content of CSAC-P-TiO_2—_NC:** The weight of an empty crucible was taken, 1.0 g of CSAC-P-TiO_2—_NC was added into it before re-weighing and heating in an oven for 5 h at 100 °C before drying in desiccators for 24 h. The weight was taken again according to [9]. The percentage of moisture was obtained from the differences in the initial and final mass of carbon using Eq. (2):
  (2) $$\text{Moisture}\;\text{Content}\;\left(\%\right)=\frac{w2}{\;w1\;}\;\times \;100$$

Where: w_1_ = initial weight, w_2_ = final weight all in (g)

iv. **Ash Content Determination of CSAC-P-TiO_2—_NC:** Empty crucible was pre-heated at 100 °C for 5 min, allowed to cool before reweighing, 1.0 g of CSAC-P-TiO_2—_NC was added to the pre-heated crucible before transferring it to a furnace to heat at 500 °C for 1½ hours. It was allowed to cool in desiccators for 24 h before re-taking the weight [9,12]. The percentage ash content was obtained using the Eq. (3):
  (3) $$\%\;\text{Ash}\;\text{content}=\frac{w1}{w2}\;\times \;100$$

Where: w_1_ = weight before drying; w_2_ = weight after oven drying.

v. **Surface area by Saer:** The surface area was elucidated by following Saer’s procedure. 0.5 g of CSAC-P-TiO_2—_NC was added to a 250 mL conical flask containing 50 mL NaCl. The pH was adjusted with 0.1 M HC and 0.1 NaOH [9]. The volume of acid or base used to cause an increase in pH from pH 4 to pH 9 was recorded, from which the surface area was calculated with Eq. (4).
  (4) $$S\left(\frac{m^{2}}{g}\right)=32v-25$$

Where; V is the volume of reagent used, S is the surface area in $\frac{m^{2}}{g}$

vi. **Volatile Matter Determination:** The weight of the empty crucible was taken, and 1.0 g of CSAC-P-TiO_2—_NC was placed on it before the weight was re-taken. The crucible was placed in a furnace to heat at 500 °C for 10 min. The final weight was taken again after cooling, as reported by [9,12]. The percentage of volatile matter was evaluated with the Eq. (5):
  (5) $$\%\text{Volatile}\;\text{matter}=\frac{w1}{w2}\times 100$$

Where: w1 = volatile matter weight (g) and w2 = oven dry weight (g)

vii. **Point of Zero Charge (pH_pzc_):** The point where the final pH and the initial pH are equal to zero is known as the point of zero charge (pHpzc). It was determined as follows: 1.0 g of CSAC-P-TiO_2—_NC was weighed into conical flasks containing 0.1 M NaCl solution. The pH of each of the flasks was adjusted from pH 2 to pH 12 with 0.1 M HCl or 0.1 M NaOH solution. The flasks were sealed while shaking for 24 h, the final pH was taken, and a plot of the differences between the initial pH and the final pH was made against the initial pH [8,13].
viii. **Iodine Number:** To elucidate the porosity of the prepared nanocomposite, the iodine number test was carried out. A standardized stock iodine solution of 100 mL was prepared, and 0.5 g of CSAC-P-TiO_2—_NC was added with continuous stirring. 100 mL of stock solution was measured into a beaker and placed on a shaker for 60 min, and then filtered. 20 mL of the filtered CSAC-P-TiO_2—_NC was titrated against 0.1 M sodium thiosulphate [9]. The amount of iodine used was calculated with Eq. (6):
  (6) $$\frac{1\;\text{mg}}{g}=\frac{B-S}{B}\times \frac{\text{VM}}{W}\times 253.81$$

*W* = mass of adsorbent. *S* = volume of thiosulphate used for the titration of the sample. *B* = volume of thiosulphate used for the titration of the blank. *V* = 20 mL aliquot. *M* = iodine solute concentration. Atomic mass of iodine = 251.83.

The porosity and surface area of the developed base material and nanocomposites from this study can be calculated using Eqs. (7) and (8):

(7) $$\text{Iodine}\;\text{value}=\frac{\text{concentration}\;\times \;\text{atomic}\;\text{mass}\;\times \;\text{normality}\;\times \;\text{volume}\;\text{of}\;\text{iodine}}{\text{mass}\;\text{of}\;\text{activated}\;\text{carbon}\;\times \;\text{volume}\;\text{of}\;\text{blank}\;\text{thiosulphate}}$$

(8) $$S_{\text{BET}}=\frac{\text{iodine}\;\text{number}\times 10^{-3}}{\text{atomic}\;\text{mass}\;\text{of}\;\text{iodine}}\times N_{A}\times \omega$$

Where: Avogadro’s number, (6.023 × 10^23^), equals N_A_, ω = 0.2096 × 10^–18^

ix. **Boehm Titration:** Boehm’s titration is used to determine acidic and basic functional groups in a sample. 1.0 g of CSAC-P-TiO_2—_NC was weighed into beakers containing 0.1 M NaOH, 0.5 M Na_2_CO_3_, 0.1 M NaHCO_3_ and 0.1 M HCl to determine the acidic and basic groups. They were placed in an electric shaker for 48 h, and back titrations were done with 0.1 M NaOH and 0.1 M HCl for basic and acidic groups, respectively [9,22]. The number of functional groups containing oxygen was determined with the Eq. (9):
  (9) $$F_{x}=\frac{Vb_{x}-V_{\text{ex}}}{M_{x}}\times M_{t}\times D_{F}$$

Where: F_x_ (mmol/g) = number of functional groups containing oxygen, D_F_ = dilution factor, M_t_ = molarity of the titrant used, V_bx_ = titrant volume used to titrate the blank and V_ex_ = titrant volume used to titrate the extract.

## Spectroscopic characterization

a. **Brunauer-Emmett-Teller (BET)/Barrett-Joyner-Halender (BJH)**

The surface area, pore size and pore volume of the prepared CSAC-P-TiO_2—_NC were examined with the Brunauer-Emmett-Teller (BET)/ Barrett-Joyner-Halender (BJH) using Brunauer Emmett Teller (BET, Quantachrome Autosorb IQ2, FL, USA).

b. **X-Ray Diffraction**

The composites (CSAC-K-TiO_2—_NC) crystalline nature was elucidated by XRD analysis [14,23]. In this study, the average size of the crystallite was calculated with the Debye-Scherer’s in Eq. (10);

(10) $$D=\frac{K\lambda }{\beta COS\theta }$$

Given that: D is the crystalline size, K is grain shape factor (for spherical grain shape, it is approximately 0.94), λ is the wavelength of the X-ray (0.1504), θ is diffraction angle at FWHM (full width at half maximum) expressed in radians.

c. **Molecular Environment Determination by Fourier Transform Infrared (FTIR) Spectroscopy**

The molecular functional components of CSAC-P-TiO_2—_NC were determined using Attenuated Total Reflection-Fourier Transform Infrared (ATR-FTIR) spectroscopy (Bruker Optics, Germany), equipped with a single-reflection Ge crystal attenuated total reflectance (ATR, Pike technologies), with measurements conducted in the range of 4000–400 cm^-1^

d. **Thermogravimetric analysis (TGA)**

The thermal stability of the prepared CSAC-P-TiO_2—_NC was performed by carrying out Thermogravimetric analysis (TGA) using thermogravimetric analysis (TGA, Q500, USA) under an air atmosphere from room temperature to 1000 °C with a heating rate of 10 °C/min.

e. **Transmission Electron Microscopy (TEM)**

The particle sizes and the general morphology of the prepared nanocomposite were determined with the Transmission Electron Microscopy (TEM) using high-resolution transmission electron microscopy (HRTEM, JEOL JEM-2100F) at 200 keV equipped with a GATAN 894 US1000 camera.

f. **X-ray Photoelectron Spectroscopy (XPS)**

The elemental composition and binding energy of the nanocomposite were determined using the X-ray Photoelectron Spectroscopy (XPS) equipment with high-resolution X-ray photoelectron spectroscopy (HRXPS ESCALAB Xi+ Thermo-Fisher Scientific, USA)

g. **Scanning Electron Microscopy (SEM) Equipped with Energy Dispersive X-ray (EDX)**

The surface morphology of prepared CSAC-P-TiO_2—_NC was visualized by SEM analysis using a field-emission scanning electron microscope (FESEM, Thermo Fisher Verios 460 L, USA) equipped with an Oxford Instrument energy dispersive-X-ray.

## Method validation

### Physicochemical characterization

**Fig. 1:**
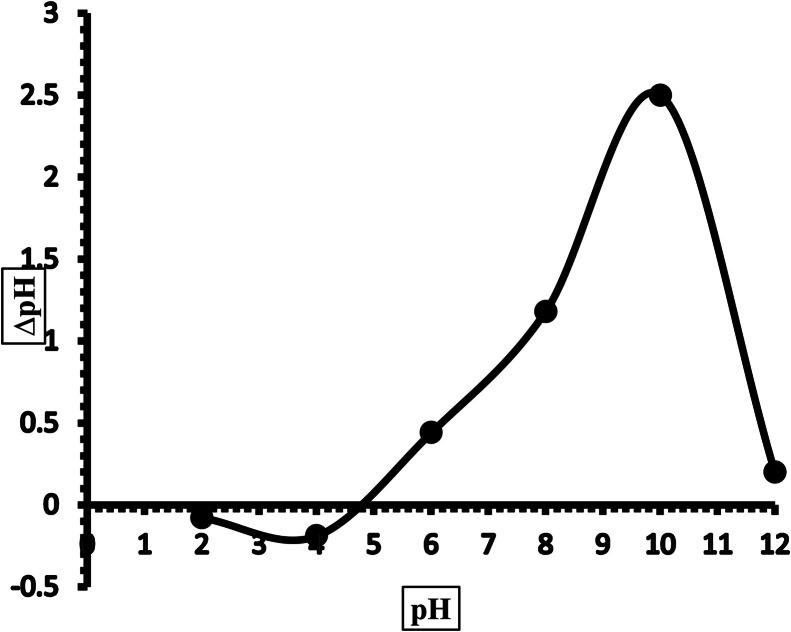
graph of pH_pzc_ of CSAC-P-TiO_2—_NC.

**Fig. 2:**
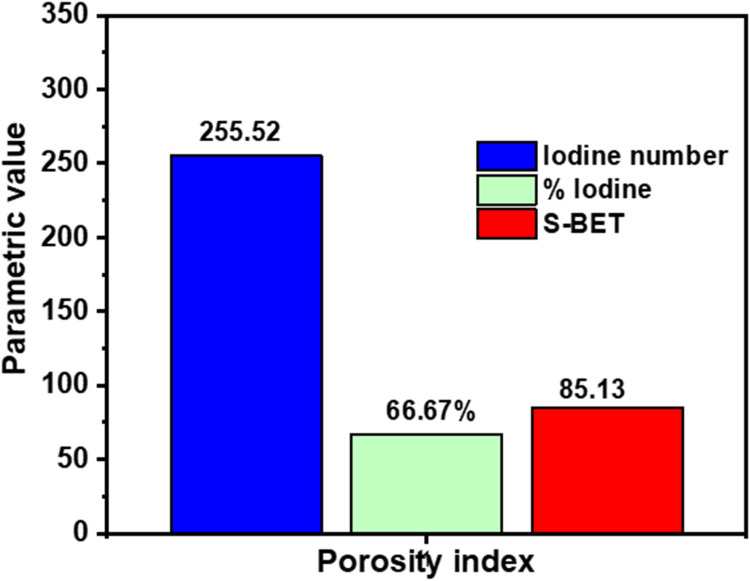
Porous properties of CSAC-P-TiO_2—_NC.

A summary of the results of the physicochemical characterization carried out on the nanocomposite is presented in Table 1. Fig. 1 is the graph of the point of zero charge. At pH greater than pH_pzc,_ the overall charge on the surface of a material is negative, while at pH lower than the pH_pzc,_ the overall charge on the material is positive. From the results obtained, the pH was greater than the pH_pzc_, which implies that the surface of the nanocomposite was negatively charged [8]. A knowledge of the charge on the surface is important in determining the suitability for application. Low ash and moisture content were obtained for the prepared CSAC-P-TiO_2—_NC that may be attributed to the heating step during preparation, which may have driven off volatile matter and moisture that may have been trapped with the molecules. The low values obtained for ash and moisture content imply that there are spaces available for pollutant uptake. Large and bulky molecules like iodine are used to estimate the removal efficiency of potential adsorbents [24,25]. The amount of iodine uptake by prepared materials gives an idea about the porous nature of that material; a high iodine number signifies the presence of large pores. Fig. 2 shows the porous properties of CSAC-P-TiO_2—_NC; the iodine number of 355.32% was obtained, suggesting the existence of large pores in the CSAC-P-TiO_2—_NC. The bulk density and the volatile matter were determined to be 0.929 and 49.4%, respectively. The bulk density gives an idea about the floatability and filterability of a given material. It is important as it affects the compactness and filterability of a material [2], and it helps in understanding if the prepared nanocomposite will float when submerged in water. Lower bulk density implies easy floatability, whereas a high bulk density suggests stability of a material that does not float easily [14,23]. The Boehm’s titration is used to estimate the total number of acidic and basic groups in a substance. From Table 1, it can be inferred that the surface of CSAC-P-TiO_2—_NC contains more acidic groups than basic groups [3].

**Table 1:** Physicochemical characterization of CSAC-P-TiO_2—_NC.

Parameter	CSAC-P-TiO_2—_NC
pH	6.57
pH_pzc_	4.8
Ash content (%)	5.3
Surface area by Saer (m^2^/g)	80.6
Moisture content (%)	5.4
Volatile content (%)	49.4
Bulk density (g/cm^3^)	0.929
Boehm titration	Acidic group: 0.1621
	Basic group: 0.0413

## Surface area and pore size determination by BET

Large specific surface area obtained by the BET machine (St 3 on NOVA touch 3LX [s/n: 17,016,110,501) was 85.130 mg^2^/g, while the BJH data revealed the surface area to be 40.7874 m²/g, pore volume 0.0843128 mL/g, pore radius Dv (r)1.88191 nm and pore size of 3.764 nm, confirming the existence of mesoporous surface. This high surface, which corroborates the surface area obtained by the simple Saer’s method, is indicative that the nanocomposite has a very promising potential and will be suitable for applications in processes such as adsorption requiring wide surface areas.

### X-ray diffraction (XRD)

The CSAC-P-TiO_2—_NC nanocomposites' crystalline nature was elucidated by XRD analysis [26], as presented in and CSAC-K-TiO_2—_NC) as shown in Fig. 2. In this study, the average size of the crystallite was calculated with the Debye-Scherer’s equation

(11) $$D=\frac{K\lambda }{\beta COS\theta }$$

**Fig. 3:**
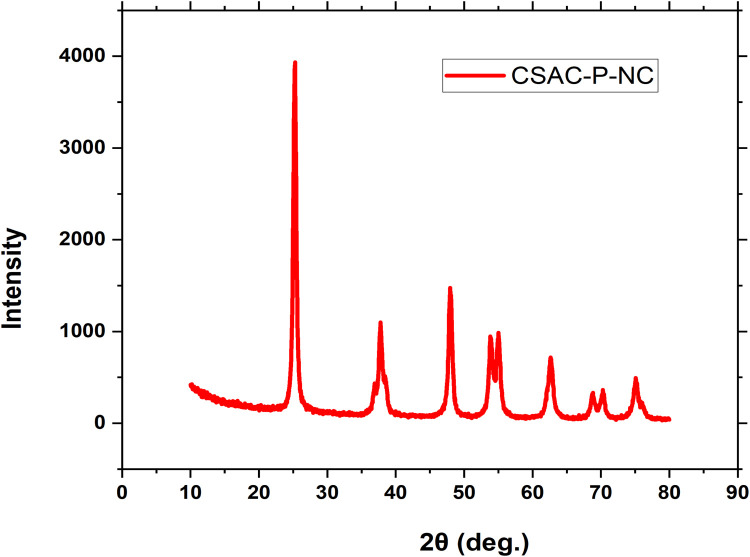
XRD pattern of CSAC-P-TiO_2—_NC.

Given that: D is the crystalline size, K is the grain shape factor (for spherical grain shape, it is approximately 0.94), λ is the wavelength of the X-ray (0.1504), θ is the diffraction angle at FWHM (full width at half maximum) expressed in radians. The crystalline size calculated in this study averaged 14.64 nm. XRD patterns of CSAC-P-TiO_2—_NC showed in Figs. 3, indicate a high amount of titanium dioxide was loaded on the acid-activated CSAC-P. The XRD patterns of CSAC-P-TiO_2_ showed intense diffraction peaks at 2θ = 25.3° which tallies with the JDPSC card number 21 −1272 and (101) diffraction plane, confirming titanium dioxide in anatase phase [16,17,27]. Other peaks were observed at 2θ equals 37.7, 47.9, 53.8 and 62.8 degrees corresponding to diffraction planes of [004], [200], [105] and [204]. The diffraction peaks tally with the JCPDS card number 21–1272 [16,18,19].

## Fourier transform infrared (FTIR) spectroscopy (FTIR)

Presented in Fig. 2 and Table 2 are the FTIR spectra and peaks obtained for CSAC-P-TiO_2—_NC before and after loading the CSAC with titanium dioxide nanoparticles. After loading with titanium dioxide nanoparticles, a prominent peak was observed at 799 cm^-1^ that indicates the Ti-O stretching band [19,28] reported a similar observation in literature where the modification of titanium dioxide resulted in IR peaks around 800 cm^-1^ to 450 cm^-1^. The peak at 3879 cm^-1^ corresponds to the O—H stretching band [25] which may be due to the dispersion medium of orthophosphoric acid, peaks observed at between 2325 cm^-1^ are assigned to the *C* = *C* of alkene, peaks at 1087 - 1568 cm^-1^ correspond to C—O bend of alcohol, ether or carboxylic acid [10]. A remarkable observation is that some peaks disappeared while some other new peaks emerged, and others became broader or sharper due to the interaction between the dispersion medium.

**Table 2:** FTIR peaks of CSAC-P-TiO_2—_NC and suggested functional groups.

FTIR Peak (cm^-1^)	Functional Group
799	Ti-O stretch
1087	C-O stretch
1568	C—O stretch
2325	*C* = *C* stretch of alkenes
3879	OH-stretch of alcohol

### Thermogravimetric analysis (TGA)

The thermal stability and behaviour of CSAC-P-TiO_2—_NC was ascertained by performing Thermogravimetric analysis (TGA) and derivative thermogravimetry (DTG) presented in Fig. 3. From the plot, loss in weight and transition in phase occurred at two different levels; the first occurred from 30 °C to 400 °C accounting for a weight loss of 0.033 mg representing 1.028% percentage loss in weight. The last weight loss occurred from 400 °C to 973.51 °C resulting in 2.922% percent loss in weight and 0.094 mg weight loss. The total weight loss at the end of the analysis was 3.95% percent 3.94 mg loss in weight of CSAC-P-TiO_2—_NC. At higher temperature above 500 °C, an increase in weight loss was observed which may be attributed to the evaporation of moisture trapped within interstitial axis of the nanocomposite [9]. The overall weight loss was <5.0% and lower than that obtained for the nanocomposite of zinc oxide reported by [22].

### X-ray photoelectron spectroscopy (XPS)

**Fig. 4:**
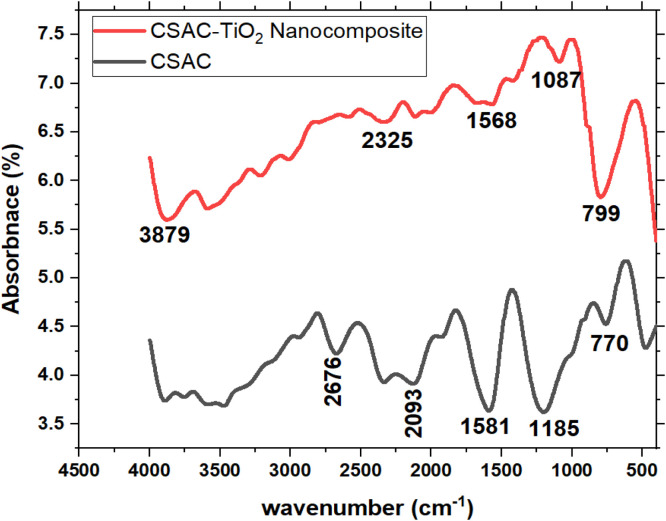
FTIR spectrum of CSAC-P before and after loading with TiO_2_ nanoparticles.

Here, XPS analysis was further conducted to investigate the chemical states and surface elemental composition of CSAC-P-TiO₂-NC, as shown in Fig. 4. The full survey spectrum clearly confirms the presence of C, O, and Ti, demonstrating the successful formation of CSAC-P-TiO₂-NC (Fig. 4a), which is consistent with the EDX results. The C 1 s spectrum exhibited three distinct characteristics: binding peaks at 284.8 eV, 286.1 eV, and 289.2 eV, which were recognised as *C* = *C*/C—C, C—O, and O—*C* = *O*, respectively (Fig. 4b). This result confirms the presence of graphitic carbon and carboxyl groups in CSAC-P-TiO₂-NC. The presence of C—O and O—*C* = *O* bonds in demonstrates abundant oxygen-containing functional groups, demonstrating partial surface oxidation of CSAC-P-TiO₂-NC. The **O 1** **s spectrum** was deconvoluted into two binding peaks centred at **530.6** **eV and 532.3** **eV (**Fig. 4c). The binding peak at 530.6 eV is assigned to **lattice oxygen (O²⁻)** in metal–oxygen bonds (M–O, where *M*=Ti), confirming the formation of a metal oxide framework. In addition, the binding-energy peak at 532.3 eV, assigned to M–OH species, is associated with surface hydroxyl groups and adsorbed oxygen species and may overlap with oxygen contributions from C–O and O–*C* = *O* bonds, indicating surface hydroxylation and interactions with carbonaceous species [29,30]. The **Ti 2p spectrum** shows two well-defined peaks at **459.4** **eV and 465.0** **eV**, corresponding to **Ti 2p₃/₂ and Ti 2p₁/₂**, respectively, with a spin–orbit splitting of approximately **5.6** **eV** (Fig. 4d). These binding energies are characteristic of **Ti⁴⁺**, confirming that titanium exists predominantly in the oxidized state, consistent with the formation of **TiO₂ or Ti-based oxide phases** [29,31]. Overall, the XPS measurement confirms the successful formation of a **Ti⁴⁺-based oxide** with **lattice oxygen**, while the presence of **surface hydroxyl groups and oxygenated carbon species** suggests surface functionalization and enhanced interfacial interactions.

### Scanning electron microscopy/energy dispersive X-ray (SEM-EDX)

**Fig. 5:**
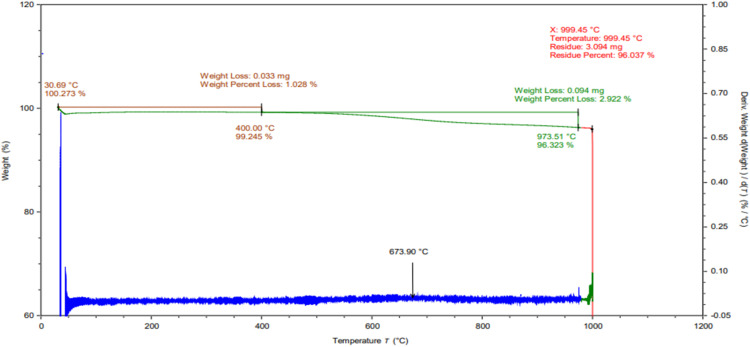
TGA and DTG analysis of CSAC-P-TiO_2—_NC.

The morphological, elemental composition, selected area electron diffraction (SAED), and electron mapping of the nanocomposite are shown in the SEM image, EDX spectrum, SAED and Electron mapping image in Fig. 5(A-D). Result revealed the elemental composition of the prepared carbon-supported titanium nanocomposite, which showed the presence of Ti, O, C and other elements [23]. The SEM image in Fig. 5A revealed the compacted morphology of CSAC-TiO_2—_NC, showing a wide range of mesoporous spaces, indicating their availability for use in trapping pollutants [9]. Fig. 5B depicts the SAED confirming the crystallinity nature of the titanium nanocomposite, Fig. 5C demonstrates the very intense titanium and oxygen peaks signifies the formation of the Ti-O bond [14,23]. Fig. 5(D) is the electron mapping displaying oxygen atoms in blue and titanium atoms in red. (Fig. 7)

### Transmission electron microscopy (TEM)

**Fig. 6:**
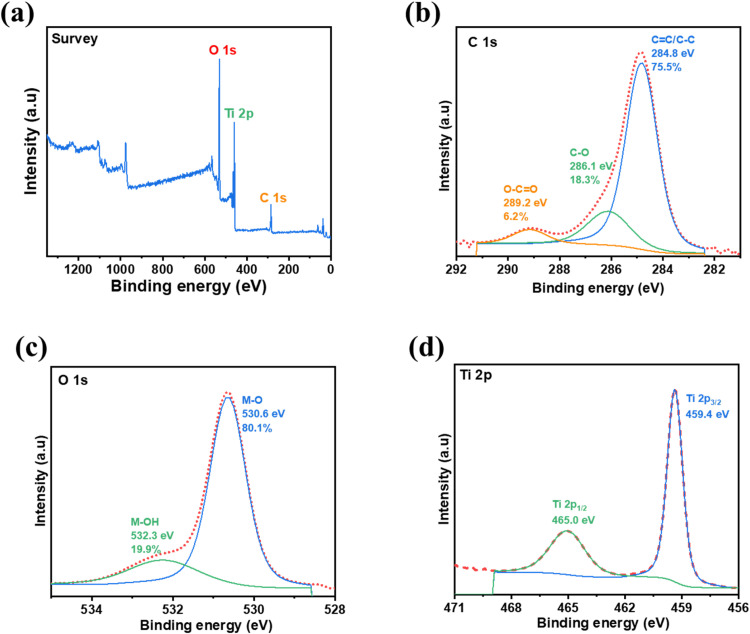
XPS spectra depicting (a) XPS survey of C, Ti & O, (b) C 1 s spectrum, (c) O 1 s spectrum and (d) Ti 2p.

Fig. 6 (A-F) presents the micrograph images of TEM analysis at different magnifications. TEM analysis is an indispensable tool in the characterization of nanomaterials; it is significant in elucidating the shapes and sizes of nanomaterials [32]. The surface morphology of the CSAC-P-TiO_2—_NC is shown in Fig. 4 (A-F). The shape of the nanocomposite was spherical, and the size was determined to be 55–60 nm by using the Image J application software. Images in Fig. 4(C) depict the fine anatase crystalline mesoporous structure of the titanium dioxide nanocomposites. (Fig. 8)

**Fig. 7:**
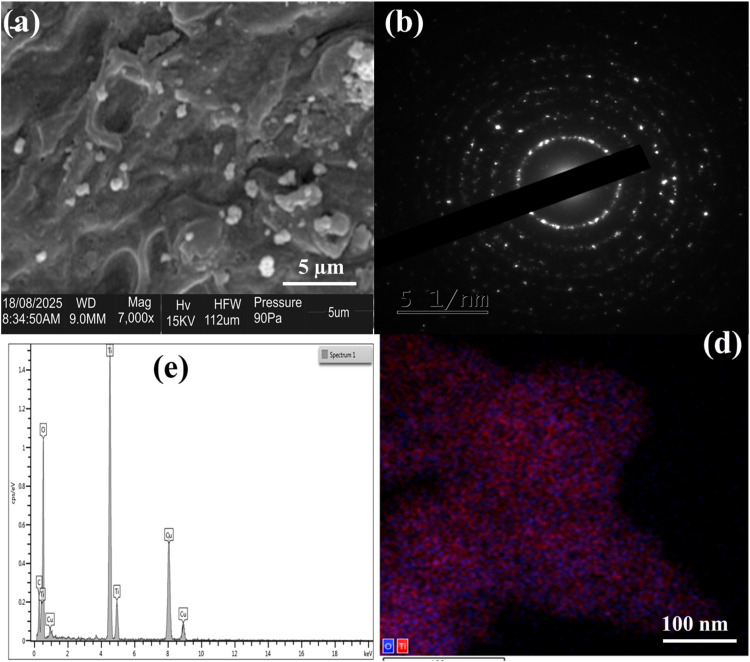
(A) SEM Morphology (B) Crystallinity (C) EDX spectrum and (D) Electron mapping of CSAC-P-TiO_2—_NC.

**Fig. 8:**
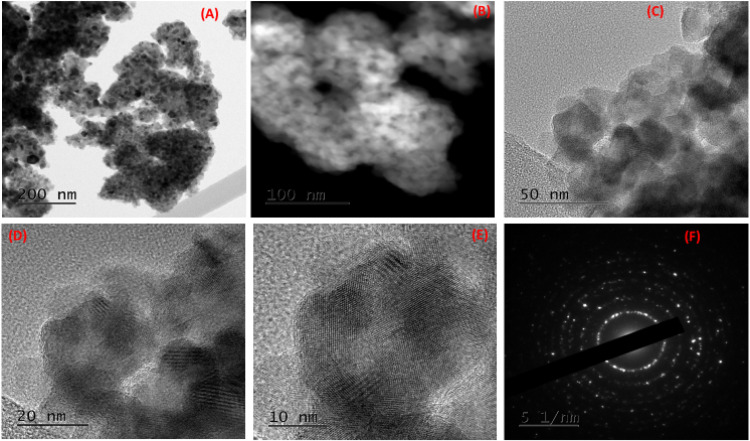
(A – F) High Resolution TEM images of CSAC-TiO_2_–NC.

## Conclusions

This study has successfully demonstrated the preparation and extensive characterization of novel coconut shell activated carbon (CSAC) supported titanium dioxide nanocomposite (CSAC-P-TiO_2—_NC) through a cost-effective and sustainable approach. Through complementary physicochemical, structural, morphological, and spectroscopic analyses, the integration of anatase-phase TiO_2_ into phosphoric acid-activated CSAC was confirmed, validating the successful formation of a stable carbon decorated nanocomposite. The physicochemical analyses demonstrated favourable development of surface properties (pH 6.57, pHpzc 4.8, 85.130 m²/g surface area), low ash content, and about 79.69% of the acidic group sites confirmed by Boehm titration. FTIR, XRD, SEM, TEM, TGA, and XPS collectively verified the incorporation of anatase TiO₂ (55–60 nm) within a mesoporous carbon matrix and its excellent thermal stability up to 900 °C. The synergistic structural and surface characteristics of CSAC-P-TiO₂-NC highlight it as a promising, low-cost, and environmentally sustainable adsorbent and photocatalyst recommended for wastewater remediation applications.

## CRediT author statement

**Adewumi Oluwasogo Dada**: Conceptualization, Methodology, Supervision, Reviewing and Editing

**Oladipo A Christianah**: Supervision, Data validation and *Re*-editing;

**Olowojoba-Ozoh M Odufa**: Data curation, Writing- Original draft preparation, Project investigation, Software

**Kehinde S Obayomi**: Resources, Visualization and Data Validation, Writing- Reviewing and Editing

**Ajanaku O. Christiana**: Visualization and Data Validation

**Sook K Chang**: Resources, Editing and Data Validation

**Ling S Wong**: Resources, Editing and Data Validation

**Lee D Wilson**: Visualization, Data Validation and Editing

## Related research article

A. O Dada, A.A. Inyinbor, B.E. Tokula, A. Bayode, K.S Shola Obayomi, Christiana Oluwatoyin Ajanaku, Folahan Amoo Adekola, Kolawole Oluseyi Ajanaku, Ujjwal Pal (2024). Zinc oxide decorated plantain peel activated carbon for adsorption of cationic malachite green dye: Mechanistic, kinetics and thermodynamics modelling, Environmental Research, Volume 252, Part 3, 2024, 119,046, https://doi.org/10.1016/j.envres.2024.119046.

A.O. Dada, F. A. Adekola, E.O. Odebunmi, F.E. Dada, O. S. Bello, A. S. Ogunlaja, Bottom-up approach synthesis of core-shell nanoscale zerovalent iron (CS-nZVI): Physicochemical and spectroscopic characterization with Cu(II) ions adsorption application, MethosX 7 (2020) 100,976. https://doi.org/10.1016/j.mex.2020.100976

## Declaration of competing interest

The authors declare that they have no known competing financial interests or personal relationships that could have appeared to influence the work reported in this paper.

## Data Availability

Data will be made available on request.
